# The link between low maternal cholesterol levels and increased risk of fetal patent ductus arteriosus and septal defects: Large-scale cohort retrospective study

**DOI:** 10.12669/pjms.42.8.19548

**Published:** 2026-08

**Authors:** Zhaodong Liu, Shuhua Lai, Guangcheng Xing, Caihong Jiang, Yulong Zhang, Jianying Yan

**Affiliations:** 1 Zhaodong Liu Department of Obstetrics, College of Clinical Medicine for Obstetrics & Gynecology and Pediatrics, Fujian Medical University, Fujian Maternity and Child Health Hospital, Fuzhou, Fujian Province 350001, P.R. China; 2 Shuhua Lai Department of Neonatology, College of Clinical Medicine for Obstetrics & Gynecology and Pediatrics, Fujian Medical University, Fujian Maternity and Child Health Hospital, Fuzhou, Fujian Province 350001, P.R. China; 3 Guangcheng Xing Department of Obstetrics, College of Clinical Medicine for Obstetrics & Gynecology and Pediatrics, Fujian Medical University, Fujian Maternity and Child Health Hospital, Fuzhou, Fujian Province 350001, P.R. China; 4 Caihong Jiang Department of Medical Ultrasonic, College of Clinical Medicine for Obstetrics & Gynecology and Pediatrics, Fujian Medical University, Fujian Maternity and Child Health Hospital, Fuzhou, Fujian Province 350001, P.R. China; 5 Yulong Zhang Department of Obstetrics and Gynecology, College of Clinical Medicine for Obstetrics & Gynecology and Pediatrics, Fujian Medical University, Fujian Maternity and Child Health Hospital, Fuzhou, Fujian Province 350001, P.R. China; 6 Jianying Yan Department of Obstetrics, College of Clinical Medicine for Obstetrics & Gynecology and Pediatrics, Fujian Medical University, Fujian Maternity and Child Health Hospital, Fuzhou, Fujian Province 350001, P.R. China

**Keywords:** Congenital heart diseases, Cholesterol, Dyslipidemia

## Abstract

**Objective:**

To investigate whether low maternal cholesterol levels during pregnancy are associated with an increased risk of neonatal Patent Ductus Arteriosus (PDA) and Atrial or Ventricular Septal Defect (ASD/VSD).

**Methodology:**

Medical records of 36,633 pregnant women who received care and delivered at Fujian Maternity and Child Health Hospital, China, from 2016 to 2020 were retrospectively analyzed. Maternal serum lipid levels were measured one week before delivery, and Congenital heart disease (CHD) was diagnosed by echocardiography within three days of birth. Logistic regression models were adjusted for neonatal sex, maternal age, gestational diabetes and hypertension, gravidity, parity, and preterm birth.

**Results:**

Among 36,633 mother-child pairs, 906 (2.5%) cases of CHD were identified. Lower maternal TC, LDL-C, and HDL-C levels were significantly associated with increased CHD risk. Each 1 mmol/L increase in TC was associated with an 11% decrease in CHD risk (OR: 0.89, 95% CI 0.85, 0.95). LDL-C showed a similar protective effect (OR: 0.88, 95% CI 0.82, 0.95), while HDL-C demonstrated the strongest association (OR: 0.61, 95% CI 0.51, 0.73). The relationship between TG and CHD risk was not significant after adjustment (OR: 0.97, 95% CI 0.92, 1.02; p=0.224). For PDA specifically, TC, LDL-C, and HDL-C remained significantly protective; for ASD/VSD, only TC demonstrated significance (OR: 0.91, 95% CI 0.83, 0.99). TG showed no significant association with any CHD subtype.

**Conclusion:**

This study indicates that lower maternal levels of TC, LDL-C, and HDL-C may be significantly associated with an increased risk of PDA, while lower TC levels could be associated with ASD/VSD risk. The relationship between maternal TG levels and CHD could not be demonstrated. Future prospective multicenter and intervention studies are needed to validate the results.

## INTRODUCTION

Congenital heart diseases (CHD), including patent ductus arteriosus (PDA) and atrial/ventricular septal defect (ASD/VSD), are seen in about nine of 1,000 live-born neonates.^1^,^2^ A PDA is defined as failure of the ductus arteriosus (DA) to close within 72 hours after birth. This fetal vessel allows in-utero access of oxygenated blood from the placenta bypassing fetal lungs, to close within 72 hours after birth.^3^ DA, which is not closed after 72 hours, is associated with substantial infant morbidity and mortality^4^ due to several complications like pulmonary edema, necrotizing enterocolitis, and congestive heart failure. Septal defects are the most common congenital cardiac defect and result from abnormal septum formation during embryonic heart morphogenesis. Moreover, large open VSDs may lead to pulmonary arterial hypertension and an increased risk of arrhythmias.^5^

Studies have shown that the nutritional status of pregnant women and the levels of lipids, including cholesterol and triglycerides, have an important role in fetal cardiac development.^6^,^7^ However, most studies have focused on the effect of high lipid levels on fetal heart development, and not on low lipid levels. During early pregnancy, fetal cholesterol synthesis is limited, leading to a high dependence on maternal cholesterol for development.^8^ Maternal total cholesterol (TC) and low-density lipoprotein (LDL) levels change modestly during pregnancy, while high-density lipoprotein (HDL) levels rise in the second trimester before reducing in the third.^9^

These fluctuations are important for the formation of fetal cell membranes and for energy, which are critical for proper heart development.^10^ Nevertheless, there is still no consensus on optimal maternal cholesterol levels during pregnancy, with some research showing that excessive TC levels may be linked with adverse pregnancy outcomes.^11^ While recent research indicates a potential association between maternal dyslipidemia and elevated CHD risk in offspring, results remain inconclusive.^12^ Therefore, this study aimed to investigate whether low maternal cholesterol levels are associated with the risk of neonatal CHD in offspring.

## METHODOLOGY

This retrospective cohort study was conducted at the Fujian Maternity and Child Health Hospital, China. Women who delivered singleton infants between January 2016 and December 2020 were included. Of 44,703 clinical records screened, 8,070 were excluded for missing records, maternal comorbidities (e.g., diabetes, cardiopathy), gestational age <24 or >44 weeks, twin pregnancy, premature PDA, or parental history of CHD. Finally, 36,633 women: 35,727 in the normal group and 906 in the CHD group were eligible, further divided into PDA (n=478), ASD/VSD (n=339), and other CHD (n=89).

### Ethical approval:

The study protocol was approved by the hospital ethics committee in accordance with the Declaration of Helsinki (No. 2024KY051, Date: March 27, 2024), with informed consent waived by the committee.

### Collected indexes:

Lipid profiles were examined as per literature.^13^ Maternal serum TG, TC, HDL-C, and LDL-C were measured from fasting blood samples collected one week before delivery using an automatic biochemistry analyzer (Abbott Architect C16000, USA). Duplicate testing was performed on 5% of samples. Reference ranges were: HDL-C 1.29–1.55 mmol/L; LDL-C 0-3.12 mmol/L; CHOL 0-5.2 mmol/L; TG 0-1.7 mmol/L.

CHD was diagnosed based on echocardiography performed within three days after birth. Two or more specialists independently examined the echocardiograms. CHD cases were categorized into PDA, ASD/VSD, and other CHDs based on International Classification of Diseases codes. ASD and VSD were grouped together as they shared embryological origin in septal morphogenesis, and there were a limited number of cases for separate subgroup analysis. PDA was defined as ductus remaining open within three days after birth. Temporary neonatal conditions and infants with trisomies, heterotaxy syndrome, or abnormal cardiac looping were excluded from the analysis^14^. All examinations were performed strictly for clinical diagnostic purposes.

### Statistical analysis:

R version 4.1.0 was used for the analysis. We summarized continuous variables as mean ± standard deviation or median (interquartile range), and categorical variables as frequency (percentage). Group differences were examined by one-way ANOVA with Tukey’s post-hoc test for normally distributed variables and the Kruskal–Wallis test with Dunn’s post-hoc test for non-normally distributed variables (triglycerides). Categorical variables were compared using the chi-square test. Associations between lipid profiles and CHD were examined using binary logistic regression for overall CHD and multinomial logistic regression for CHD subtypes, adjusted for neonatal sex, maternal age, gestational diabetes, gestational hypertension, gravidity, parity, and preterm birth.

## RESULTS

In Table-I, the baseline data of the study is included. Mothers of infants with PDA had significantly lower TC levels (5.8 ± 1.2 mmol/L) compared to mothers of normal infants (6.1 ± 1.2 mmol/L). This trend was consistent across all lipid profiles, including TG, LDL, and HDL. Mothers of infants with CHDs were more likely to be older (18.3% vs. 14.9% elderly parturients), and gestational diabetes and hypertension were more prevalent, especially with PDA. Infants with CHD had a significantly higher incidence of prematurity (38%) compared to normal neonates (9.4%), with PDA showing the highest rate (43.1%) (Table-I).

**Table-I:** Characteristics of the cohort.

Variables	Total (n = 36,633)	Normal (n = 35,727)	CHD				P value
			All CHD (n = 906)	PDA (n = 478)	ASD/VSD (n = 339)	Other (n = 89)	
Maternal age, Mean ± SD	30.5 ± 4.5	30.5 ± 4.5	30.8 ± 5.0	31.1 ± 5.0	30.7 ± 5.0	29.4 ± 4.9	0.100
TC, Mean ± SD	6.1 ± 1.2	6.1 ± 1.2	5.9 ± 1.2	5.8 ± 1.2***	5.9 ± 1.3**	5.9 ± 1.2	< 0.001
TG, Median (IQR)	3.1 (2.4, 4.0)	3.1 (2.4, 4.0)	2.9 (2.4, 3.8)	2.9 (2.4, 3.8)	2.9 (2.3, 3.9)	3.0 (2.5, 4.2)	0.006
LDL-C, Mean ± SD	3.0 ± 0.9	3.0 ± 0.9	2.9 ± 0.8	2.8 ± 0.8***	2.9 ± 0.9	2.9 ± 0.9	< 0.001
HDL-C, Mean ± SD	1.8 ± 0.4	1.8 ± 0.4	1.7 ± 0.4	1.6 ± 0.4***	1.7 ± 0.4**	1.7 ± 0.4*	< 0.001
Elderly parturient, n (%)							0.005
No	31157 (85.1)	30417 (85.1)	740 (81.7)	381 (79.7)	280 (82.6)	79 (88.8)	
Yes	5476 (14.9)	5310 (14.9)	166 (18.3)	97 (20.3)	59 (17.4)	10 (11.2)	
Multipara, n (%)							0.167
No	20505 (56.0)	19977 (55.9)	528 (58.3)	295 (61.8)	180 (53.1)	52 (58.4)	
Yes	16128 (44.0)	15750 (44.1)	378 (41.7)	182 (38.2)	159 (46.9)	37 (41.6)	
Gravidity, n (%)							0.175
1	14705 (40.1)	14356 (40.2)	349 (38.5)	204 (42.8)	112 (33)	32 (36)	
2	10672 (29.1)	10419 (29.2)	253 (27.9)	122 (25.6)	101 (29.8)	30 (33.7)	
≥3	11256 (30.7)	10952 (30.7)	304 (33.6)	151 (31.7)	126 (37.2)	27 (30.3)	
Gestational diabetes mellitus, n (%)							< 0.001
No	29754 (81.2)	29096 (81.4)	658 (72.6)	338 (70.7)	253 (74.6)	67 (75.3)	
Yes	6879 (18.8)	6631 (18.6)	248 (27.4)	140 (29.3)	86 (25.4)	22 (24.7)	
Gestational hypertension, (%)							< 0.001
No	34886 (95.2)	34061 (95.3)	825 (91.1)	431 (90.2)	309 (91.2)	85 (95.5)	
Yes	1747 (4.8)	1666 (4.7)	81 (8.9)	47 (9.8)	30 (8.8)	4 (4.5)	
Premature birth, n (%)							< 0.001
No	32921 (89.9)	32359 (90.6)	562 (62)	272 (56.9)	225 (66.4)	65 (73)	
Yes	3712 (10.1)	3368 (9.4)	344 (38)	206 (43.1)	114 (33.6)	24 (27)	
Sex of newborn infant, n (%)							0.373
1(Male)	19437 (53.1)	18970 (53.1)	467 (51.5)	242 (50.6)	174 (51.3)	51 (57.3)	
2(Female)	17196 (46.9)	16757 (46.9)	439 (48.5)	236 (49.4)	165 (48.7)	38 (42.7)	

* p < 0.05,

** p < 0.01,

*** p < 0.001 for comparisons between the normal group and CHD subgroups, determined by one-way ANOVA with Tukey’s post-hoc test ASD/VSD: atrial or ventricular septal defect; CHD: congenital heart defect; PDA: patent ductus arteriosus; TC: total cholesterol; TG: triglyceride; LDL-C: low density lipoprotein cholesterol; HDL-C: high density lipoprotein cholesterol.

The results of a binary logistic regression analysis assessing the relationships between maternal lipid profiles and CHD risk shows in Table-II. The study included 36,633 mother-child pairs, with 906 (2.5%) cases of CHD. Both unadjusted and adjusted analyses showed a significant inverse relationship between TC and CHD risk. The analysis showed that adjusted odds of CHD decreased by 11% per 1 mmol/L increase in TC (OR 0.89, 95% CI 0.85-0.95, p<0.001). Higher LDL was also associated with lower CHD risk (OR 0.88, 95% CI 0.82-0.95, p=0.002). The strongest association was seen with HDL (OR 0.61, 95% CI 0.51-0.73, p<0.001). The marginally significant unadjusted relationship between TG and CHD risk disappeared after adjustment.

**Table-II:** Binary logistic regression analysis of the relationships between lipids profiles and congenital heart defect.

Variable	Total	CHD	Crude		Adjusted	
		n (%)	OR (95%CI)	P value	OR (95%CI)	P value
TC, mmol/L	36,633	906 (2.5)	0.83 (0.78~0.88)	<0.001	0.89 (0.85~0.95)	<0.001
TG, mmol/L			0.95 (0.90~1.00)	0.043	0.97 (0.92~1.02)	0.224
LDL-C, mmol/L			0.83 (0.77~0.90)	<0.001	0.88 (0.82~0.95)	0.002
HDL-C, mmol/L			0.43 (0.36~0.52)	<0.001	0.61 (0.51~0.73)	<0.001

TC: total cholesterol; TG: triglyceride; LDL-C: low density lipoprotein cholesterol; HDL-C: high density lipoprotein cholesterol. Adjusted for infant sex, maternal age, gestational diabetes mellitus, gestational hypertension, gravidity, parity, and premature birth.

Table-III shows a multinomial logistic regression analysis of maternal lipid profiles and CHD subtypes. PDA showed the strongest associations: higher TC, LDL, and HDL were all significantly associated with lower risk, with each 1 mmol/L increase in HDL decreasing the risk of PDA. For ASD/VSD, only TC showed a significant association. However, for other CHDs, only HDL was significant. Given the small number of cases in the other CHD subgroup (n=89), this finding should be interpreted cautiously. No significant associations were noted between TG and any CHD subtype. The protective effect of higher maternal cholesterol levels was strongest for PDA.

**Table-III:** Multinomial logistic regression analysis of the associations between lipids profiles and specific congenital heart defect subtypes.

Variable	PDA			ASD/VSD			Other^#^		
	n (%)	OR (95%CI) vs. Normal	P value	n (%)	OR (95%CI) vs. Normal	P value	n (%)	OR (95%CI) vs. Normal	P value
TC, mmol/L	478 (1.3)	0.88 (0.82~ 0.95)	0.001	339 (0.9)	0.91 (0.83~ 0.99)	0.040	89 (0.2)	0.89 (0.75~ 1.06)	0.200
TG, mmol/L		0.96 (0.90~ 1.03)	0.229		0.94 (0.87~ 1.02)	0.159		1.11 (0.98~ 1.27)	0.102
LDL, mmol/L		0.84 (0.76~ 0.94)	0.002		0.94 (0.83~ 1.06)	0.331		0.86 (0.67~ 1.10)	0.242
HDL, mmol/L		0.50 (0.39~ 0.65)	<0.001		0.82 (0.61~ 1.09)	0.173		0.52 (0.29~ 0.91)	0.023

ASD/VSD: atrial or ventricular septal defect; CHD: congenital heart defect; PDA: patent ductus arteriosus; TC: total cholesterol; TG: triglyceride; LDL-C: low density lipoprotein cholesterol; HDL-C: high density lipoprotein cholesterol;

# : Congenital heart defect other than atrial or ventricular septal defect, and patent ductus arteriosus.

## DISCUSSION

This study showed that pregnant women delivering healthy neonates had overall higher levels of TC, TG, LDL-C, and HDL-C than women with infants diagnosed with CHD. Lower levels of TC, LDL-C, and HDL-C were associated with an increased risk of CHD, particularly PDA, in offspring. The association between TG and CHD became non-significant after adjustment for confounders. Abnormal maternal lipid levels may therefore impair infant heart development, increasing the risk of CHD, especially PDA.

Throughout pregnancy, TC increases to meet the demands of fetal growth, owing to its role in steroid hormone synthesis.^15^-^17^ Our results detected significantly lower TC, LDL-C, and HDL-C in women with PDA (ORs 0.50-0.88, all P<0.005), while TG showed no such association; lower TC levels were also linked to ASD/VSD risk. Earlier studies have linked both high^18^ and low^19^-^21^ maternal cholesterol levels to adverse outcomes such as preterm birth, low birth weight, and intrauterine growth restriction. Such conflicting results may reflect different mechanisms: prior studies linking hyperlipidemia to CHD largely focused on hyperglycemia-induced dyslipidemia or severe hypercholesterolemia, where excess lipids may promote oxidative stress or endothelial dysfunction in the fetal heart.^22^

However, cholesterol is an essential substrate for fetal cell membrane synthesis and steroid hormone production, and cholesterol-dependent signaling pathways, such as Hedgehog signaling, which may be related to septation and ductal remodeling.^23^-^25^ It is therefore plausible that lipid–CHD associations are “U-shaped”: both extreme hypercholesterolemia and hypocholesterolemia may increase risk, while moderate cholesterol levels support optimal fetal cardiac development. We may speculate that low cholesterol levels increased the risk of preterm birth, consequently leading to the observed higher rates of PDA.

Although the observed intergroup differences in lipid levels were minimal, even slight differences in maternal cholesterol may have meaningful developmental impacts, as fetal cholesterol requirements are high during critical cardiac development windows such as weeks 4–8 of gestation, when the ductus arteriosus and ventricular septum form. In 2018, Lee et al.^25^showed that lowering cholesterol levels by statins in early pregnancy significantly increased the risk of CHD. Our study demonstrated that for every one mmol/L increase in HDL-C, PDA risk decreased by 50%, a large effect size that, if validated in prospective study, could contribute to future risk stratification strategies.

Existing research suggests that elevated cholesterol levels during the third trimester increase the risk of preterm birth (PB)^26^; however, 12.7% of women with low total cholesterol also had preterm deliveries, and low cholesterol often indicates poor nutrition and predicts PB and low birth weight.^27^ PDA is the most common cardiovascular disease in infants who are small for gestational age, with extremely premature infants facing a PDA risk of approximately 80% at three days.^3^,^28^

We may hypothesize that low maternal cholesterol levels increase the risk of PDA and ASD/VSD through two potential mechanisms: impaired remodeling of the ductus arteriosus that prevents postnatal closure, or disruption of cholesterol-dependent cardiac gene expression governing septation and morphogenesis.^29^ PDA is likely influenced by a combination of genetic and environmental factors. Importantly, consistent with recent studies^22^, this paper shows that even lower maternal cholesterol levels during the third trimester may affect the maturation of smooth muscle and endothelial function in the fetal ductus arteriosus; however, this proposed mechanism remains speculative, as no molecular or genetic data were collected in this study. The closure of the ductus arteriosus depends on the contractile function of smooth muscle cells.^30^ As cholesterol is a key precursor of membrane components and vasoactive substances, abnormal cholesterol levels in the third trimester may interfere with the signal pathway for ductus arteriosus closure.

### Strength of the study:

Unlike previous reports that primarily examined early-pregnancy lipid levels or hyperlipidemia, this study focuses on the less-explored impact of relatively low maternal cholesterol measured in late pregnancy. While prior studies largely assessed CHD as a composite outcome^30^, our analysis systematically evaluates distinct CHD subtypes using a large, single-center cohort (n=36,633) with standardized lipid measurement and echocardiographic confirmation of CHD, providing sufficient power to detect subgroup-specific associations, particularly for PDA and ASD/VSD.

### Limitations:

The study excluded patients who received prenatal care and delivered singleton infants between January 2021 and February 2024, due to the launch of a new medical information system in our two campuses. This exclusion was due to incomplete digital records during the system migration rather than any clinical selection criterion, and we have no reason to believe it introduced systematic bias relevant to the lipid–CHD associations examined. The research focused only on TG, TC, HDL-C, and LDL-C rather than broader lipid profiles or additional risk factors such as apolipoproteins. Because lipid levels were measured near term rather than during weeks 4–8 of gestation, when ductal and septal morphogenesis occurs, the present findings may not capture the lipid exposure most relevant to cardiac development, limiting causal interpretation. While statistically significant differences were observed between the normal and CHD groups, the actual values were quite close, suggesting possible reverse causality or measurement error. Undiagnosed fetal CHD could theoretically alter maternal lipid metabolism via placental dysfunction, though exclusion of fetuses with major structural anomalies minimizes this risk.

Measurement error was minimized using standardized fasting lipid assays and duplicate testing (coefficient of variation <5%). Excluding pregnancies under 24 weeks may have introduced bias, and despite known associations between low cholesterol and poor nutrition,^21^ no TC-BMI correlation was assessed. The study did not adjust for maternal BMI, nutritional status, socioeconomic factors, or lifestyle. The CHD group also had a significantly higher preterm birth rate (38% vs. 9.4%), and we did not perform mediation analyses to explore the role of preterm birth in the lipid-CHD relationship. Given the close relationship between prematurity and PDA, the observed lipid-PDA association may be partially driven by gestational age at birth rather than an independent effect of maternal lipid levels.

### Clinical implications:

This study suggests the need to modify the scope of prenatal surveillance, as maternal cholesterol may be a determinant of fetal cardiovascular health rather than merely a metabolic parameter. The possible association between low maternal cholesterol and increased PDA/ASD-VSD risk suggests a need for a more balanced approach to lipid management during pregnancy, since insufficient cholesterol may be as detrimental as excess. Hypocholesterolemia may represent a potential obstetric risk factor. Given the small absolute differences in lipid levels between groups, these findings should be regarded as hypothesis-generating rather than as a basis for immediate changes to clinical practice.

## CONCLUSION

This study indicates that lower maternal levels of TC, LDL-C, and HDL-C may be significantly associated with an increased risk of PDA, while lower TC levels could be associated with ASD/VSD risk. The relationship between maternal TG levels and CHD could not be demonstrated. Further research is needed to identify women at high risk and to determine whether interventions can improve outcomes.

### Authors’ contributions:

**ZL and SL:** Literature search, study design and manuscript writing.

**GX, CJ, YZ and JY:** Data collection, data analysis and interpretation. Critical review.

**ZL and SL:** Manuscript revision and validation and is responsible for the integrity of the study.

All authors have read and approved the final manuscript.
